# A network analysis of exhaustion disorder symptoms throughout treatment

**DOI:** 10.1186/s12888-024-05842-9

**Published:** 2024-05-23

**Authors:** Gustav Mårtensson, Fred Johansson, Monica Buhrman, Fredrik Åhs, Jakob Clason van de Leur

**Affiliations:** 1 Department of Psychology, Uppsala University, Box 1225, Uppsala, 751 42 Sweden; 2 Department of Health Promotion Science, Sophiahemmet University, Valhallavägen 91, Stockholm, SE-114 28 Sweden; 3 Department of Psychology and Social Work, Mid Sweden University, Kunskapens väg 1, Östersund, SE-831 40 Sweden

**Keywords:** Exhaustion due to persistent non-traumatic stress, Stress-induced exhaustion disorder, Exhaustion disorder, Clinical burnout, Network analysis, Network theory, Network connectivity

## Abstract

**Background:**

Stress-induced Exhaustion Disorder (ED) is associated with work absenteeism and adverse health outcomes. Currently, little is known regarding how the symptoms of ED are interrelated and whether the patterns of symptoms influence treatment outcomes. To this end, the current study applied network analyses on ED patients participating in a multimodal intervention.

**Methods:**

The first aim of the study was to explore the internal relationships between exhaustion symptoms and identify symptoms that were more closely related than others. A second aim was to examine whether the baseline symptom network of non-responders to treatment was more closely connected than the baseline symptom networks of responders, by comparing the sum of all absolute partial correlations in the respective groups’ symptom network. This comparison was made based on the hypothesis that a more closely connected symptom network before treatment could indicate poorer treatment outcomes. Network models were constructed based on self-rated ED symptoms in a large sample of patients (*n* = 915) participating in a 24-week multimodal treatment program with a 12-month follow-up.

**Results:**

The internal relations between self-rated exhaustion symptoms were stable over time despite markedly decreased symptom levels throughout participation in treatment. Symptoms of limited mental stamina and negative emotional reactions to demands were consistently found to be the most closely related to other ED symptoms. Meanwhile, sleep quality and irritability were weakly related to other exhaustion symptoms. The symptom network for the full sample became significantly more closely connected from baseline to the end of treatment and 12-month follow-up. The symptom network of non-responders to treatment was not found to be more closely connected than the symptom network of responders at baseline.

**Conclusions:**

The results of the current study suggest symptoms of limited mental stamina and negative emotional reactions to demands are central ED symptoms throughout treatment, while symptoms of irritability and sleep quality seem to have a weak relation to other symptoms of ED. The implications of these findings are discussed in relation to the conceptualization, assessment, and treatment of ED.

**Trial registration:**

The clinical trial was registered on Clinicaltrials.gov 2017-12-02 (Identifier: NCT03360136).

## Background

Work-related stress and exhaustion, commonly reported by workers, are associated with work absenteeism and adverse health outcomes [1–4]. The costs of work-related stress to Western societies have been estimated to be as high as 187 billion dollars due to losses in productivity and medical expenses [5]. Patients with exhaustion due to persistent non-traumatic stress as their primary complaint report various somatic and psychiatric symptoms, and there is a lack of international consensus on how this heterogeneous condition should be diagnosed and understood [6–10]. In Sweden, the diagnostic criteria of stress-induced Exhaustion Disorder (ED) (SE-ICD-10; F43.8 A) are utilized to diagnose and guide the treatment of exhaustion due to persistent non-traumatic stress. The ED diagnosis is characterized as a reaction to a prolonged period of persistent non-traumatic stress, resulting in a lack of psychological energy and an increased need for recovery, and for many, symptoms of cognitive deficiencies, irritability, poor sleep quality, and several somatic symptoms [11]. The prevalence of ED has increased rapidly since its introduction in 2005, and it is today one of the most common psychiatric disorders in Sweden, raising some concerns about the current diagnostic conceptualization of ED [12, 13]. Regardless of the specific diagnostic construct of ED, there is an apparent need to increase the understanding of the symptoms that underlie this condition, in Sweden and globally [6, 7, 14].

The network theory of mental disorders is a new perspective on mental illness which challenges the traditional assumption that psychiatric symptoms are caused by distinct underlying disorders [15]. Instead, network theory proposes that mental disorders could develop and be maintained by complex causal relationships between the psychiatric symptoms themselves [15–17]. Considering the heterogeneous nature of exhaustion due to persistent non-traumatic stress, perhaps the network theory could improve the understanding of the condition by focusing on interactions between symptoms of exhaustion rather than attempting to understand it as a discrete disease entity.

Studies based on the network theory have largely used statistical network analysis methods to estimate and visualize complex internal relations between symptoms involved in a disorder [18]. Indices of network centrality have been used to discover symptoms that are closely interrelated with other symptoms in the network [19, 20]. For example, researchers have found that fatigue is a centrally connected symptom of depression, along with the DSM-V depression symptoms of loss of interest, depressed mood, and concentration problems [21, 22]. These four central symptoms are strongly correlated with other depressive symptoms and are also the best predictors for the onset of major depression [22]. Identifying central symptoms may be of clinical importance, as it has been suggested that these symptoms could represent important treatment targets [17, 18].

Network connectivity, the sum of all absolute partial correlations in a symptom network corresponding to a disorder, has been suggested to influence symptom severity and the longitudinal course of psychiatric disorders [18, 23]. The underlying assumption of this hypothesis is that higher connectivity suggests stronger reinforcing relationships between symptoms, causing disorders to persist [18]. Some studies have retroactively found a lower baseline network connectivity in symptom networks for groups of patients who later improved from depression, compared to those who did not improve [23–25]. However, other studies have found no association between baseline network connectivity and treatment response [26, 27].

The current study used network analyses in a large clinical sample of ED patients, to explore the internal relationships between ED symptoms. Our first aim was to investigate the centrality of ED symptoms to identify symptoms that can potentially play a key role in maintaining ED and thus represent important treatment targets. Our second aim was to determine if there were differences in the pre-treatment network connectivity between responders and non-responders to treatment, as predicted by the connectivity hypothesis.

## Method

### Design and participants

Participants data was collected as part of an open clinical trial of a 24-week multimodal intervention (MMI) with a 12-month follow-up. The study was conducted at two healthcare centres (PBM Sweden AB) in Stockholm, Sweden, from October 2017 through December 2020. The clinical trial was approved by the Regional Ethical Review Board in Stockholm (Approval Nr. 2016/1834- 31/2) and followed the ethical principles of the Declaration of Helsinki. All participants provided written consent before inclusion.

The participants were recruited via referrals from healthcare services in the Stockholm area. Inclusion criteria were (1) an ED diagnosis confirmed through assessment by a team of three different clinicians (a licensed psychologist, a licensed physiotherapist, and an M.D.), (2) a self-rated score of at least 4.5 points on the Shirom-Melamed Burnout Questionnaire [28], and (3) age 18 to 64. Exclusion criteria were (1) substance abuse, (2) moderate-high suicidal risk, and (3) severe psychiatric illness (for example severe schizophrenia, untreated PTSD or bipolar disorder). The participants’ use of medication was not restricted. The sample includes data from 915 participants. The 24-week MMI was a standardized multidisciplinary treatment based on a cognitive behavioural model. It includes various components, for example, individual and group-based CBT, applied relaxation, medical treatment, physical exercise, physiotherapy, and return-to-work planning. Four articles have previously been published based on other aspects of this data, more specifically on symptom and return-to-work outcomes, sub-groups, predictors of improvement and construct validity of the Karolinska Exhaustion Disorder Scale (KEDS) [29–32].

The participants answered surveys, which included self-rating of ED symptoms, at five separate time points throughout the trial, spanning roughly 19.5 months in total: (1) at the initial assessment, (2) the start of treatment (≈ 1.5 months following assessment), (3) halfway through treatment (3 months following the start of treatment), (4) the end of treatment (6 months following the start of treatment), (5) at a 12-months follow-up (following the end of treatment).

### Measurements

ED symptoms were measured using the Karolinska Exhaustion Disorder Scale (KEDS). KEDS is a self-rating questionnaire with nine questions, which are rated on a seven-point scale ranging from zero to six points, with a total sum score ranging from 0 to 54. A total score of 19 or above indicates “at risk of ED” [33]. Cronbach’s alpha for KEDS at the initial assessment of the sample was 0.75.

Each item in KEDS represents a specific ED symptom corresponding to the Swedish diagnostic criteria. The items, along with their respective zero- and six-point choices, are [33]: Ability to concentrate (*I do not have any difficulty concentrating, and can read, watch TV and converse normally; I cannot concentrate on anything at all*). Memory (*I remember names, dates, and what I am supposed to do; Every day, I forget important things or what I have promised to do*). Physical stamina (*I feel the way I usually do and perform my daily physical activities or exercise as usual; I feel very weak and cannot even move short distances*). Mental stamina (*I have just as much energy as usual. I do not have any particular difficulty performing my daily activities; I do not have the energy to do anything*). Recovery (*I do not have to rest during the day; No matter how much I rest, it feels as if I am unable to recharge my batteries*). Sleep (*I sleep well and long enough. I usually feel thoroughly rested when I wake up after a night’s sleep; I sleep superficially or restlessly every night. I never feel thoroughly rested after a night’s sleep*). Hypersensitivity to sensory impressions (*I do not think that my senses are more sensitive than usual; Sound, light or other sensory impressions bother me so much that I withdraw in order to give my senses a chance to rest*). Experience of demands (*I do what I am supposed to do or want to do without experiencing it as especially demanding or difficult; l experience nearly everything as demanding and cannot handle it at all*). Irritation and anger (*I do not feel that I am especially easily irritated; I am often furious and have to make an enormous effort in order to restrain myself*).

The construct validity of KEDS has, however, been questioned in a recent psychometric article, which warrants further examination of the ED symptomatology as described by KEDS [32].

### Differentiating responders and non-responders

To test the hypothesis that higher network connectivity, the sum of all absolute partial correlations in the networks, could be associated with a worse treatment outcome, the participants were categorized as responders or non-responders to treatment. Treatment response was defined as fulfilling the criteria of clinically significant change [34]. To be classified as responders, participants had to (1) show a reduction in KEDS score from the initial assessment to the 12-month follow-up of 9 points or more, which indicates a reliable change [34], and (2) score below the cut-off of 19 points on KEDS at the 12-month follow-up [33]. This resulted in a responder group of 328 participants and a non-responder group of 455. Due to missing data at the 12-month follow-up, 132 participants could not be assigned to either group.

### Statistical analysis

#### Network estimation and stability

**Fig. 1:**
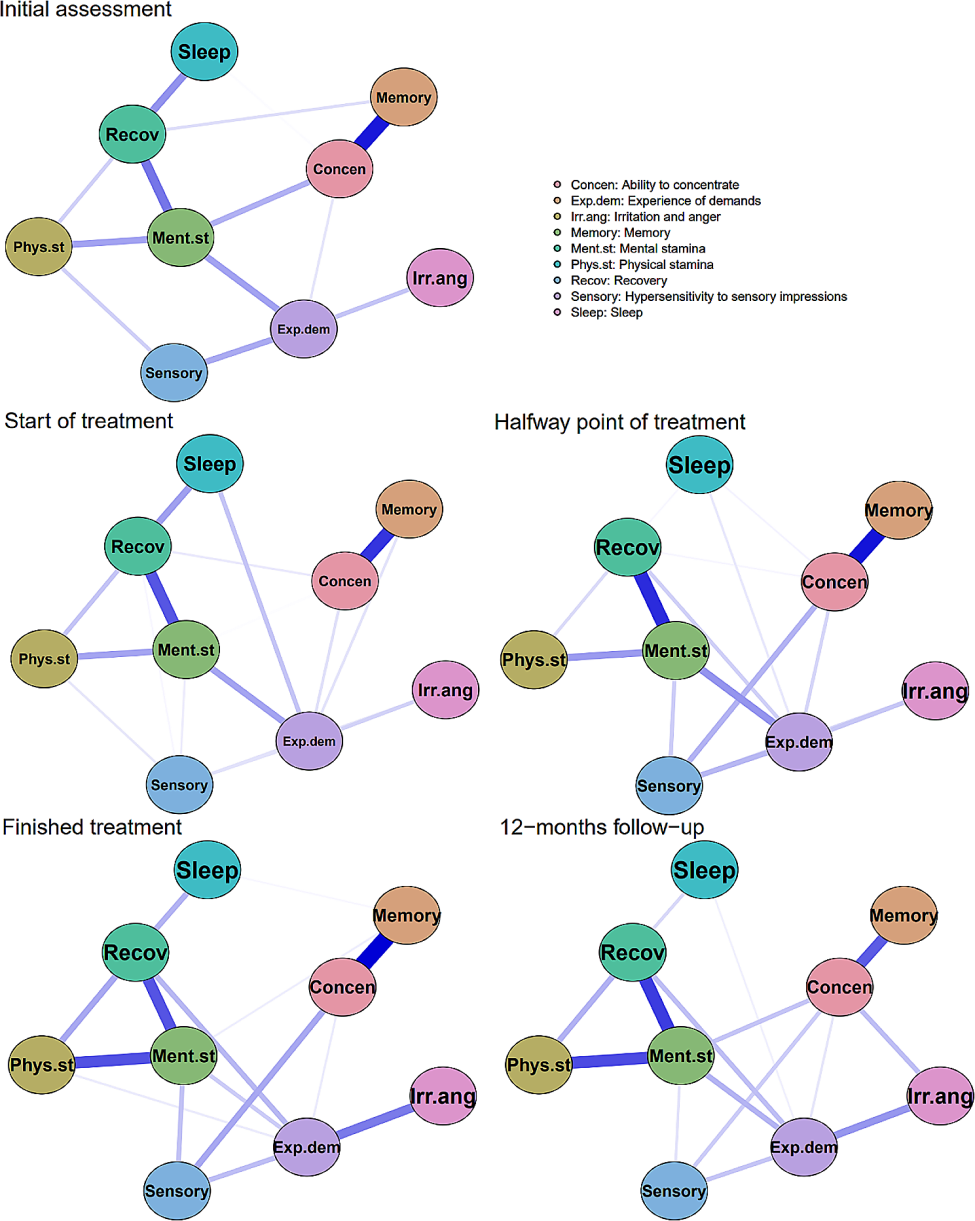
Unregularized partial correlation networks for all time points of KEDS self-rating. The visual layout based on self-ratings at the initial assessment is used for all networks. Blue edges represent positive partial correlations. Edge weights with an absolute value below ρ = 0.1 are omitted. The size and saturation on all visual edges are relative to the maximum edge weight in any of the networks (ρ = 0.37) and represent the strength of the partial correlations

**Fig. 2:**
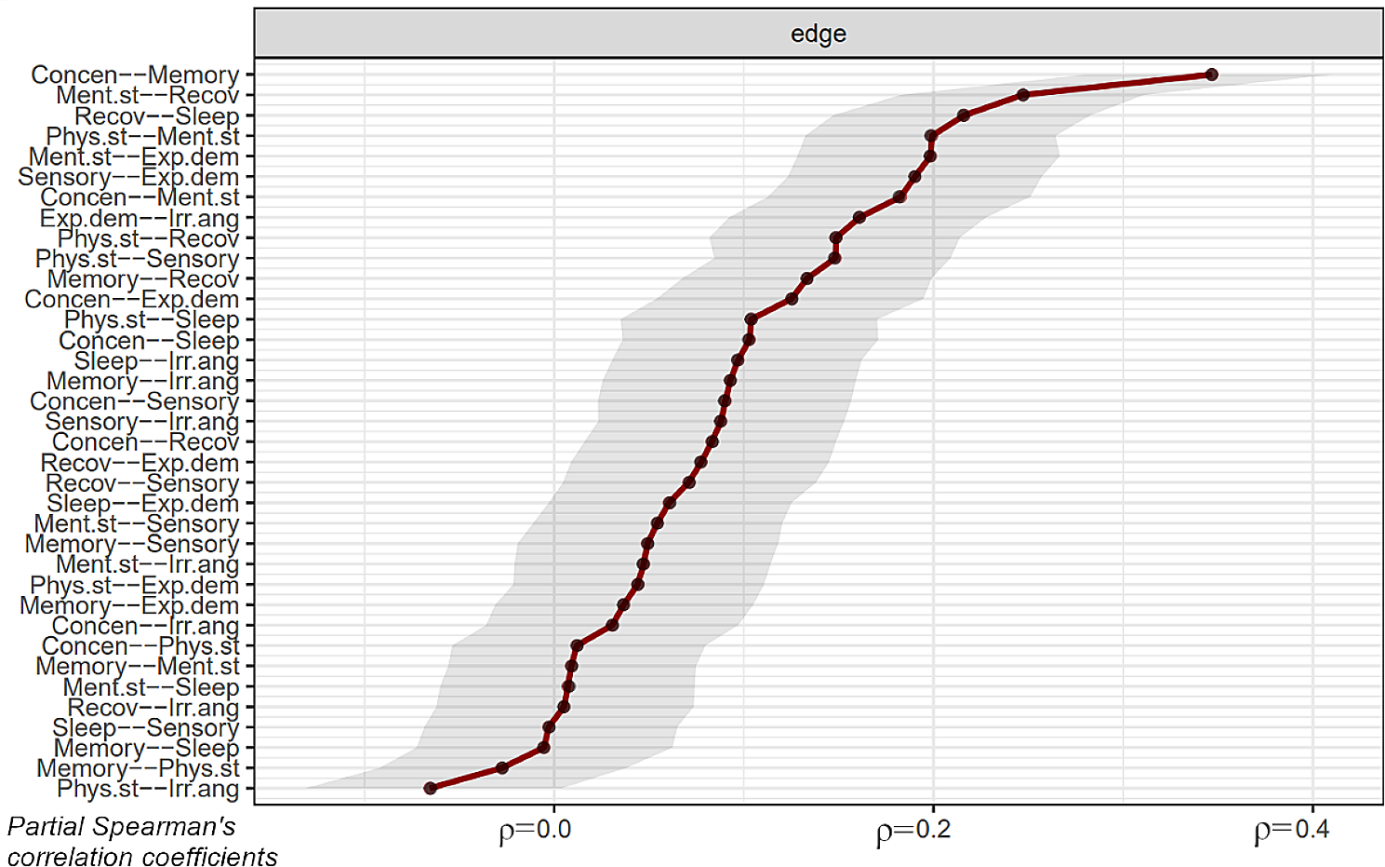
Edge weights for the network at the initial assessment and the variation of the bootstrap estimates of edge weights. The x-axis shows the partial Spearman´s correlation coefficient. The overlapping red and black lines indicate the initial assessment’s edge weights and the bootstrapped samples’ average edge weights, respectively. The shaded area represents 95% confidence intervals around the edge weights

Unregularized weighted networks of the KEDS items at each time point were estimated using partial Spearman correlation matrices (Fig. 1). Confidence intervals around the edge weights for the network at the initial assessment were estimated using a non-parametric bootstrap with 10,000 samples (Fig. 2) [35, 36].

**Fig. 3:**
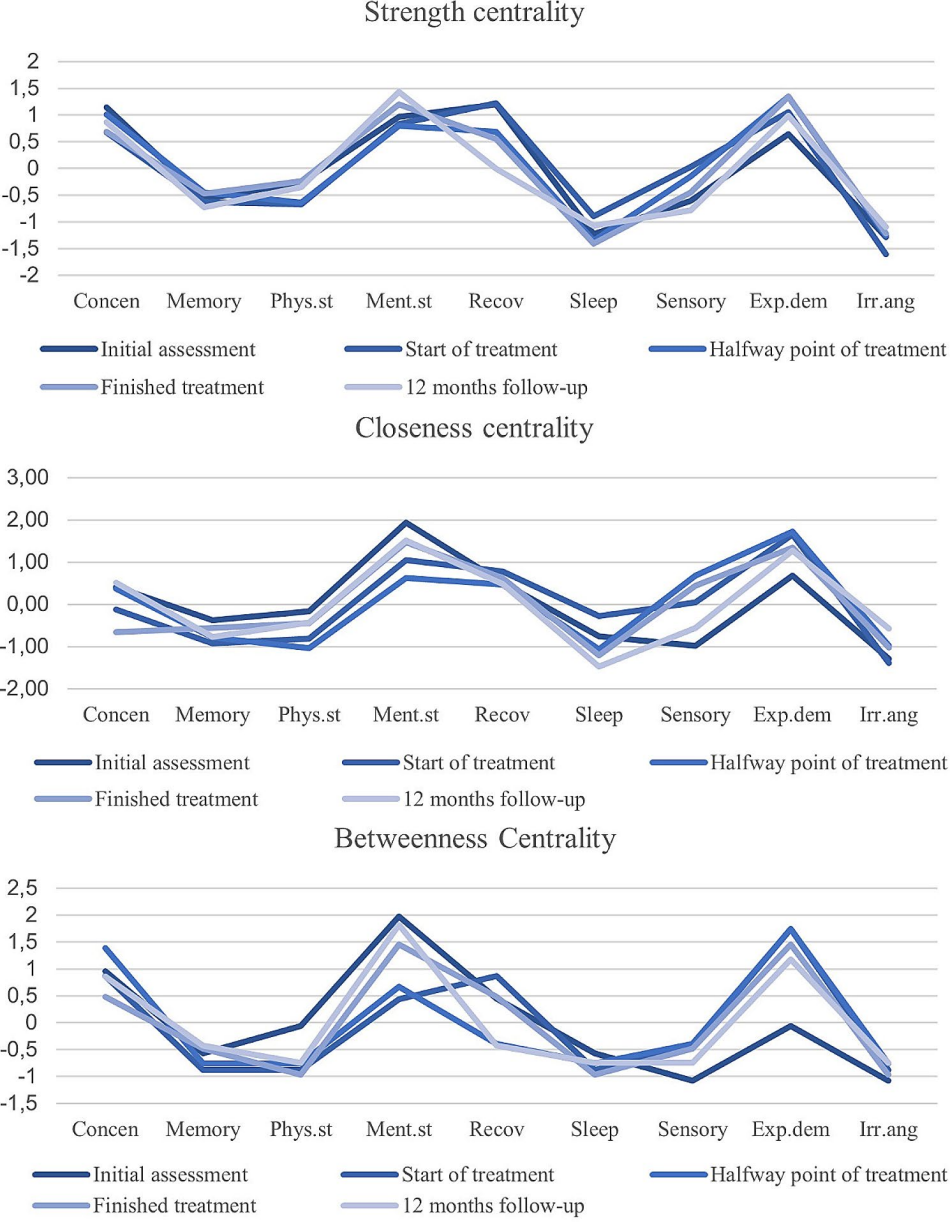
Strength, closeness and betweenness centrality for ED symptoms at all time points. The numbers on the y-axis represent the z-values of centrality indices. Concen: ability to concentrate, phys.st: physical stamina, ment.st: mental stamina, recov: recovery, sensory: hypersensitivity to sensory impressions, exp.dem: experience of demands, irr.ang: irritation and anger

Node centralities for symptoms in all networks were estimated using the centrality indices strength (the sum of absolute partial correlations connected to a node); closeness (the inverse average length of the shortest distance to all other nodes); betweenness centrality (number of times the node is located on the shortest distance between two other nodes) (Fig. 3) [19, 37]. The stability of the centrality indices for the network at the initial assessment was analysed using case-dropping bootstrap, generating 10,000 bootstrap samples consisting of subsets of the original data [36]. The results were summarized using correlation stability coefficients, indicating what proportion of the data that could be removed while retaining a correlation of at least *r* = 0.7 with the centrality indices from the original network, and are presented with a 95% confidence interval. A correlation stability coefficient of 0.5 or above is considered stable [36]. All analyses were complete case analyses, using the data available at each respective time point. This was deemed appropriate given the low attrition rate.

#### Network visualization

The visualized networks consist of nine nodes, each representing an item of the KEDS, which are connected by weighted edges, each representing partial Spearman correlation. To limit the number of edges and increase the interpretability of the visual networks, edge weights below ρ = 0.1 are hidden in the visualization of the networks. The width and saturation of edges reflect the strength of the partial correlation between nodes, in proportion to the maximum correlation in any of the networks (ρ = 0.37).

The placement of the nodes in the visualization of the networks is based on the Fruchterman-Reingold algorithm that was implemented on the networks from the initial assessment and then kept constant for the following time points to increase visual comparability between networks [38]. The algorithm places nodes with strong partial correlations close to each other, and nodes with a high absolute sum of partial correlations near the middle of the visual network.

#### Comparison of the overall network structure over time

Differences between the network from the initial assessment and the network at the end of treatment, as well as the 12-month follow-up, were analysed using the Network Comparison Test [39]. The Network Comparison Test is a permutation test, that tests if a specific difference between two networks (e.g., a difference in an edge weight) is significantly different from what would be expected by repeated random rearrangement of participants between the two groups. We used the Network Comparison Test to analyse differences in both overall connectivity and individual edge weights between networks at different time points, using 10,000 permutations.

#### Comparison of network connectivity between responders and non-responders

When comparing the network connectivity of the responder and non-responder groups at baseline, the groups were matched on total KEDS scores to rule out that group differences in initial symptom levels could explain differences in connectivity or treatment outcome. A subset of participants, 207 in total, that could not be matched due to differences in group size and baseline total scores of the responder and non-responder group, were randomly selected for exclusion from the analysis. After matching, both groups consisted of *n* = 288 participants with identical baseline KEDS total scores (while still differing in individual items scores).

Unregularized networks based on Spearman partial correlations were estimated for each group, and the difference in network connectivity was then tested using the Network Comparison Test generating 10,000 randomly rearranged pairs of groups.

Networks were estimated, visualized, and compared using the R-version 4.2.1 and the packages *bootnet* [36], *qgraph* [38] and *NetworkComparisonTest* [39].

## Results

### Sample characteristics

Our sample included 915 participants who completed the initial assessment. The mean (SD) age was 43 years (9.4), 86% were women and 71% had a university education (Table 1). The follow-up rate was 99% at the start of treatment, 97% a mid-treatment, 95% after finishing treatment and 86% at the 12-month follow-up (Table 2). Mean scores on the KEDS decreased during treatment, both for the total score and the individual item scores (Table 2).

**Table 1:** Pretreatment characteristics of the sample (*N* = 915)

	Total (*n* = 915)	Responders (*n* = 328)	Non-responders (*n* = 455)
**Demographical variables**			
Age, mean (SD)	43.0 (9.4)	42.2 (9.6)	43.6 (9.4)
Women, *n* (%)	789 (86)	282 (86)	396 (87)
**Marital status**, ***n*(%)**			
-Single or other	280 (31)	91 (28)	144 (32)
-Married/living together	572 (63)	223 (68)	270 (59)
-Partner (living apart)	63 (7)	14 (4)	41 (9)
**Education**, ***n*(%)**			
- Elementary school and/or secondary school	232 (25)	72 (22)	120 (26)
- University < 3 years	142 (16)	52 (16)	71 (16)
- University ≥ 3 years	500 (55)	188 (57)	249 (55)
- Other	41 (4)	16 (5)	15 (3)
**Household income**, ***n*(%)**			
- 0–250 000 SEK/year	76 (8)	19 (6)	47 (10)
- 250 000–500 000 SEK/year	308 (34)	97 (30)	159 (35)
- 500 000–1000 000 SEK/year	391 (43)	149 (45)	190 (42)
- > 1000 000 SEK/year	140 (15)	63 (19)	59 (13)
**Percentage of working/studying full-time, n (%)**			
- 0%	507 (55)	185 (56)	250 (55)
- 1–25%	90 (10)	30 (9)	52 (11)
- 26–50%	163 (18)	54 (16)	81 (18)
- 51–75%	39 (4)	14 (4)	20 (4)
- 76–100%	116 (13)	45 (14)	52 (11)

*Note* 132 participants could not be assigned to the responder or non-responder group, due to missing data at the 12-month follow-up

**Table 2:** Average Karolinska exhaustion disorder scales scores and missing data for all time points. MMI = Multimodal intervention. The possible value of the individual items ranges between 0–6, and the total score between 0–54

Item scores	Initial assessment	Start of MMI	Half-way point of MMI	Finished MMI	12-month follow-up
Ability to concentrate, M (SD)	3.81(1.02)	3.64(1.00)	3.07(1.13)	2.49(1.24)	2.25(1.38)
Memory, M (SD)	3.84(1.28)	3.59(1.29)	3.16(1.24)	2.79(1.25)	2.53(1.28)
Physical stamina, M (SD)	3.38(1.07)	3.30(1.04)	2.79(1.10)	2.32(1.21)	2.14(1.31)
Mental stamina, M (SD)	3.94(0.89)	3.72(0.89)	3.03(0.99)	2.50(1.09)	2.28(1.24)
Recovery, M (SD)	4.52(1.10)	4.26(1.89)	3.49(1.14)	2.99(1.14)	2.74(1.36)
Sleep, M (SD)	3.95(1.44)	3.71(1.49)	2.93(1.46)	2.46(1.42)	2.34(1.55)
Hypersensitivity to sensory impressions, M (SD)	3.93(1.41)	3.74(1.35)	3.17(1.38)	2.74(1.40)	2.51(1.46)
Experience of demands, M (SD)	4.08(0.88)	3.80(0.91)	3.08(1.08)	2.47(1.18)	2.25(1.25)
Irritation and anger, M (SD)	3.35(1.40)	3.09(1.31)	2.52(1.29)	1.96(1.24)	1.91(1.35)
Total scores, M (SD)	34.81(6.17)	32.85(6.47)	27.24(7.55)	22.72(8.14)	20.95(9.04)
Missing data, n (%)	1 (< 1%)	9 (< 1%)	28 (3%)	49 (5%)	132 (14%)

### Visualization of networks

Graphical networks based on partial Spearman correlations of KEDS items for all the time points are presented in Fig. 1.

The consistently strongest edges were between memory and the ability to concentrate, and between recovery and mental stamina. Mental stamina and the experience of demands both had strong relations to other ED symptoms for most symptom networks. Irritability and sleep both lack strong relationships to other ED symptoms in most networks.

### Edge weights

Partial Spearman’s correlations for all ED symptoms based on KEDS-rating at the initial assessment are presented in Fig. 2, along with 95% confidence intervals. In general, the 95% confidence intervals around edge weights were fairly large, indicating that differences in edge weights should be interpreted with caution.

### Node centrality

Centrality indices for all the time points are presented in Fig. 3. The correlation stability coefficient at the initial assessment for strength centrality was 0.60, closeness centrality 0.52 and betweenness centrality 0.13, indicating that closeness and strength centrality were stable, while betweenness centrality was unstable.

The ability to concentrate, mental stamina, recovery and the experience of demands all had similarly high values in strength centrality. Mental stamina and the experience of demands had the highest values in betweenness centrality and closeness centrality. Irritability and sleep both had consistently low values in all indices of centrality.

### Network comparisons

#### Comparison of networks from different time points

The network at the initial assessment was compared on network structure and network connectivity using the Network Comparison Test, to the networks at the end of treatment and the 12-month follow-up, respectively. No significant differences in individual edge weights could be found between the initial assessment and the end of treatment or the 12-month follow-up. However, the network connectivity had increased from the initial assessment (3.56) to the end of treatment (3.91, *p* < 0.1) and the higher level of connectivity was also retained at the 12-month follow-up (3.90, *p* < 0.1).

#### Comparison of networks of responders and non-responders

Symptom networks based on KEDS-self rating were estimated for the matched groups consisting of 288 responders and 288 non-responders to treatment. The network connectivity at the initial assessment was 3.84 for the responder group and 3.49 for the non-responder group. This difference was not statistically significant (*p* = 0.31).

## Discussion

The present study aimed to investigate the internal structure of ED symptoms and explore the relative connectedness of its symptoms using indices of network centrality. These analyses revealed several noteworthy findings, including limited mental stamina and negative experiences of demands consistently emerging as the most central symptoms of ED within the full sample. Conversely, irritability and sleep quality were found to be the least central symptoms in the network structure. Furthermore, the network structure appeared stable throughout the study, as no differences in individual edge weights were statistically significant when comparing symptom networks from the initial assessment to the end of treatment and the 12-month follow-up. In addition, the current study also aimed to determine if ED patients who responded to an MMI had lower network connectivity before treatment but found no significant difference in network connectivity between responders and non-responders.

### Network structure and centrality

The network structure of ED symptoms was relatively stable throughout the study. While the overall network connectivity for the full sample significantly increased from the initial assessment to the end of treatment and the 12-month follow-up, no changes in any individual edge weights were found to be statistically significant. The centrality values of symptoms at different time points are also largely consistent throughout the study. The consistency in network structure throughout multiple points of self-rating and a large reduction in symptom levels suggests that the correlations between symptoms could potentially represent meaningful relationships between ED symptoms. While the network structure was stable, it should be highlighted that the initial assessment and pre-treatment networks are likely the most representative of the ED population, since they are based on self-rated ED symptoms before manipulation through MMI treatment.

Limited mental stamina and negative experiences of demands emerged as the most central and most densely connected ED symptoms. That limited mental stamina would prove to be a central symptom was perhaps unsurprising, since it is arguably the symptom that most closely represents the larger ED construct and is a cardinal criterion for diagnosis. Meanwhile, the high centrality values of experience of demands were more surprising as it is one of the least explored ED symptoms in previous research [14]. According to network theory, central symptoms may be important treatment targets, as they are believed to be drivers of a potentially pathological self-sustaining network structure [15]. Negative reactions and lacking perceived resources when faced with demands are related to aspects of perfectionism [40–42] and previous research has found perfectionistic traits and behaviours at work to be associated with exhaustion due to persistent non-traumatic stress [43, 44]. An analysis of predictors of change based on the same sample data as the current study found that participants who scored higher on perfectionistic traits also reported higher degrees of ED before treatment, and proportionally benefitted more from MMI treatment [30]. This finding, combined with the central position of negative experiences of demands in the symptom networks of the current study, suggests that perfectionistic tendencies could represent an important psychological process to target in treatment for ED patients. Consequently, future research on clinical interventions for ED would potentially benefit from focusing on methods specifically targeting perfectionism and the negative experience of demands, for example, cognitive techniques related to self-criticism and exposure in vivo to the experience of not meeting demands.

The least central symptoms in all centrality indices were sleep quality and irritability. While research on irritability in ED patients is limited, the marginal role of sleep quality was surprising as previous research has found sleep quality/insomnia to be an important predictor for the onset of ED, and improved sleep to be a key mediating variable for recovery from ED in patients participating in CBT [45–47]. Supporting the results of the current article however, a recently published psychometric article (partly based on the same sample as the current study) used confirmatory factor analysis on KEDS responses and found the irritability and sleep items to be weakly related to the unidimensional ED construct supposedly captured by KEDS [32]. Despite the weak specific relations to other symptoms and the ED construct, improved sleep has positive effects on a wide range of psychiatric complaints [48], which means that better sleep quality would likely lead to improved health regardless of the specific condition.

It is worth noting that irritability and poor sleep are highly prevalent in a wide range of psychiatric disorders and are common reactions to stressors, such as grief and trauma [21, 49]. Given the prevalence of these symptoms in mental illness in general and the weak relationship of irritability and sleep quality to other ED symptoms in the current study and the psychometric article on KEDS [32], it seems adequate to question their diagnostic relevance and specificity to ED. The lack of empirical evidence for the ED diagnostic criteria has recently been highlighted [14]. Additionally, concerns have been raised about current ED diagnostic criteria being overly inclusive, which increases the risk of overdiagnosing and biases toward categorizing other mental disorders as ED [13]. Diagnostic constructs should be discriminatory, not comprehensive illness descriptions. Considering the findings in the current study, future research should consider removing irritability and sleep quality from the diagnostic criteria and ED measurements such as KEDS as a way of increasing the specificity of the ED diagnosis and simplifying differential diagnostics.

### Network connectivity

The finding that symptom networks of responders compared to non-responders did not differ in network connectivity means that the current study could not find support for the connectivity hypothesis, which proposes that higher network connectivity could represent stronger reinforcing feedback loops between symptoms and therefore be a predictor of a worse treatment outcome [18, 23]. Interestingly the connectivity level was higher for the responder group, although this difference is uncertain as it was not statistically significant. Before analysis, the groups were matched on baseline total KEDS score, to rule out the possibility that differences in connectivity could be explained by differences in baseline symptom levels and variance. It is worth highlighting that this meant a subset of the non-responder groups with the most severe initial symptoms were not included in the analysis, which could have affected its outcome.

The connectivity hypothesis has received some empirical support from a limited number of studies [23–25]. However, a study by Elovaino et al. [26] did not find a difference in network connectivity of symptoms of depression when comparing a large clinical sample and a group of healthy controls. The fact that the network connectivity for the full sample significantly increased from the initial assessment to the end of treatment and the 12-month follow-up, as the overall symptom levels decreased, raises further questions on network connectivity as an indicator of clinical severity and treatment outcome. Several previous studies have also found an increase in network connectivity following treatment [25, 50, 51]. McElroy et al. [25] speculate that increased connectivity following treatment could suggest “positive spirals”, whereas improvement in one symptom leads to improvements in others and, thus, stronger correlations between symptoms. Another possible explanation for increased connectivity in the current study is that floor effects at the end of treatment cause the increased correlation between symptoms, due to the low average symptom levels.

### Strengths and limitations

A major strength of the current study is the quality of the sample data on which the network analyses were based. It is the largest clinical sample of self-rated ED symptoms collected to date, with multiple time points spanning roughly 1.5 years, few dropouts, and a low degree of missing data. All patients were professionally assessed by a team of clinicians before inclusion to confirm the ED diagnosis. Women and individuals with a university education were overrepresented in the sample, compared to the Swedish population. However, based on previous research and sick leave data, this is likely somewhat representative of the demographics of the ED population [11, 52]. The open clinical trial was conducted in a naturalistic setting, and the inclusion criteria were permissive regarding medications and comorbid illnesses, which should increase the external validity of our findings.

The current study has some important limitations. The study has estimated symptom networks based on large-scale group data, which risks masking considerable individual heterogeneity or sub-group differences in the data. It is unclear to what extent aggregated group-level relationships between symptoms can be generalized to individuals [53]. Another limitation is that while the network theory of mental disorders assumes causal relationships between symptoms [15], the current study´s design does not allow for conclusions on causality.

Network models based on partial correlations are sensitive to which variables are included [53]. First, failure to include all relevant factors in a network risks creating spurious connections due to confounder bias. Second, by conditioning on the potential common effects of different nodes, network analysis is also sensitive to collider bias, by which conditioning on a common effect could create spurious relationships between nodes. Networks based on ED diagnostic symptoms may be missing unidentified variables of importance in maintaining the disorder, which means there is a risk of biases. Arguably, this risk is higher when discussing ED as compared to other disorders, as the research on its diagnostic symptoms is limited and it is not clear to what extent the current diagnostic criteria of ED adequately describe the condition [14]. In future studies, network models based on symptoms of exhaustion due to persistent non-traumatic stress could include a greater variety of variables than the ED diagnostic criteria currently used in Sweden. Network models, including, for example, symptoms of anxiety and depression as well as theoretical constructs such as perfectionism, could shed further light on what processes underlie exhaustion due to persistent non-traumatic stress and how it relates to other conditions and symptoms.

It should also be emphasized that ED has been criticized for being a poorly validated diagnostic construct [13, 14]. The scope of the current article was to examine the relative internal relatedness between these diagnostic symptoms. It is not possible from the findings of the current article to draw conclusions on the validity of the diagnosis, and whether it should be understood as a diagnostic construct distinct from validated disorders with overlapping symptoms.

## Conclusion

This study found that network structure and internal relationships between ED symptoms were stable over time, despite decreasing symptom levels. Symptoms of limited mental stamina and negative reactions to demands emerged most strongly related to other ED symptoms. Meanwhile, irritability and sleep quality were weakly related to other ED symptoms. The study found no evidence supporting network connectivity as an indicator of clinical severeness or likely treatment outcome.

In conclusion, these findings do have the potential to inform future developments of treatments, assessment tools, and diagnostic conceptualizations of exhaustion due to persistent non-traumatic stress, as they highlight the centrality of limited mental stamina and negative experiences of demands as well as the limited influence of irritability and sleep quality in the symptom network of ED.

## Data Availability

The patient data analysed in the study are not openly available due to reasons of sensitivity but are available from the corresponding author upon reasonable request.
